# Lesions of Cholinergic Pedunculopontine Tegmental Nucleus Neurons Fail to Affect Cocaine or Heroin Self-Administration or Conditioned Place Preference in Rats

**DOI:** 10.1371/journal.pone.0084412

**Published:** 2014-01-21

**Authors:** Stephan Steidl, Huiling Wang, Roy A. Wise

**Affiliations:** Intramural Research Program, National Institute on Drug Abuse, National Institutes of Health/Department of Health and Human Services, Baltimore, Maryland, United States of America; Neuroscience Campus Amsterdam, VU University, Netherlands

## Abstract

Cholinergic input to the ventral tegmental area (VTA) is known to contribute to reward. Although it is known that the pedunculopontine tegmental nucleus (PPTg) provides an important source of excitatory input to the dopamine system, the specific role of PPTg cholinergic input to the VTA in cocaine reward has not been previously determined. We used a diphtheria toxin conjugated to urotensin-II (Dtx::UII), the endogenous ligand for urotensin-II receptors expressed by PPTg cholinergic but not glutamatergic or GABAergic cells, to lesion cholinergic PPTg neurons. Dtx::UII toxin infusion resulted in the loss of 95.78 (±0.65)% of PPTg cholinergic cells but did not significantly alter either cocaine or heroin self-administration or the development of cocaine or heroin conditioned place preferences. Thus, cholinergic cells originating in PPTg do not appear to be critical for the rewarding effects of cocaine or of heroin.

## Introduction

The dopaminergic neurons of the midbrain are important for the rewarding effects of cocaine [1], [2]. They receive inputs from a number of brain regions [3], [4], and they respond not only to primary rewards but also to the peripheral stimuli that predict primary rewards [5]. Forebrain dopamine levels are modulated by midbrain infusions of several neurotransmitter agonists and antagonists [6]; of these, cholinergic agents are of particular interest because of evidence linking acetylcholine with reward function in general [7] and with cocaine reward in particular [8].

There are only two known sources of cholinergic input to the midbrain dopamine neurons: the pedunculopontine tegmental (PPTg) and laterodorsal tegmental (LDTg) nuclei [9], [10]. Among the cholinergic terminals that synapse on dopamine cells, the majority make asymmetric (presumably excitatory) synapses onto dopamine cells that give rise to nucleus accumbens projections [11]. In rats and mice, electrical stimulation of the PPTg or LDTg induces a tri-phasic pattern of dopamine efflux in the dorsal striatum or nucleus accumbens, respectively, and actions of acetylcholine at nicotinic and muscarinic cholinergic receptors in the VTA and SNc each contribute to mesopontine excitation of the dopamine system [12], [13], [14], [15].

Cholinergic excitation of VTA dopamine neurons appears to be important for reward function, as rats will work for VTA microinjections of the cholinergic agonist carbachol, which can also establish conditioned-place preference [16], [17]. Acetylcholine levels in the VTA are elevated during rewarding lateral hypothalamic electrical stimulation, during consumption of food or water rewards, and during intravenous cocaine self-administration [8], [18]. Cholinergic receptor blockade in the VTA reduces the rewarding efficacy of lateral hypothalamic electrical stimulation [19] and causes compensatory increases in cocaine self-administration in trained rats [8]. While the PPTg appears to provide the source of cholinergic input to the VTA that is important for the rewarding effects of lateral hypothalamic electrical stimulations [19], the source of cholinergic input to the VTA important for the rewarding effects of cocaine is not known. We used a neurotoxin designed to selectively lesion mesopontine cholinergic neurons [20] to test the effects of cholinergic PPTg cell loss on the rewarding effects of cocaine. In parallel we examined the role of cholinergic PPTg cell loss on the rewarding effects of opiates. Our results indicate that PPTg cholinergic function is not critical for the rewarding effects of either agent.

## Results

### PPTg cholinergic cell loss failed to alter cocaine intravenous self-administration, establishment of cocaine conditioned place preference, or affect cocaine-induced locomotion

**Figure 1:**
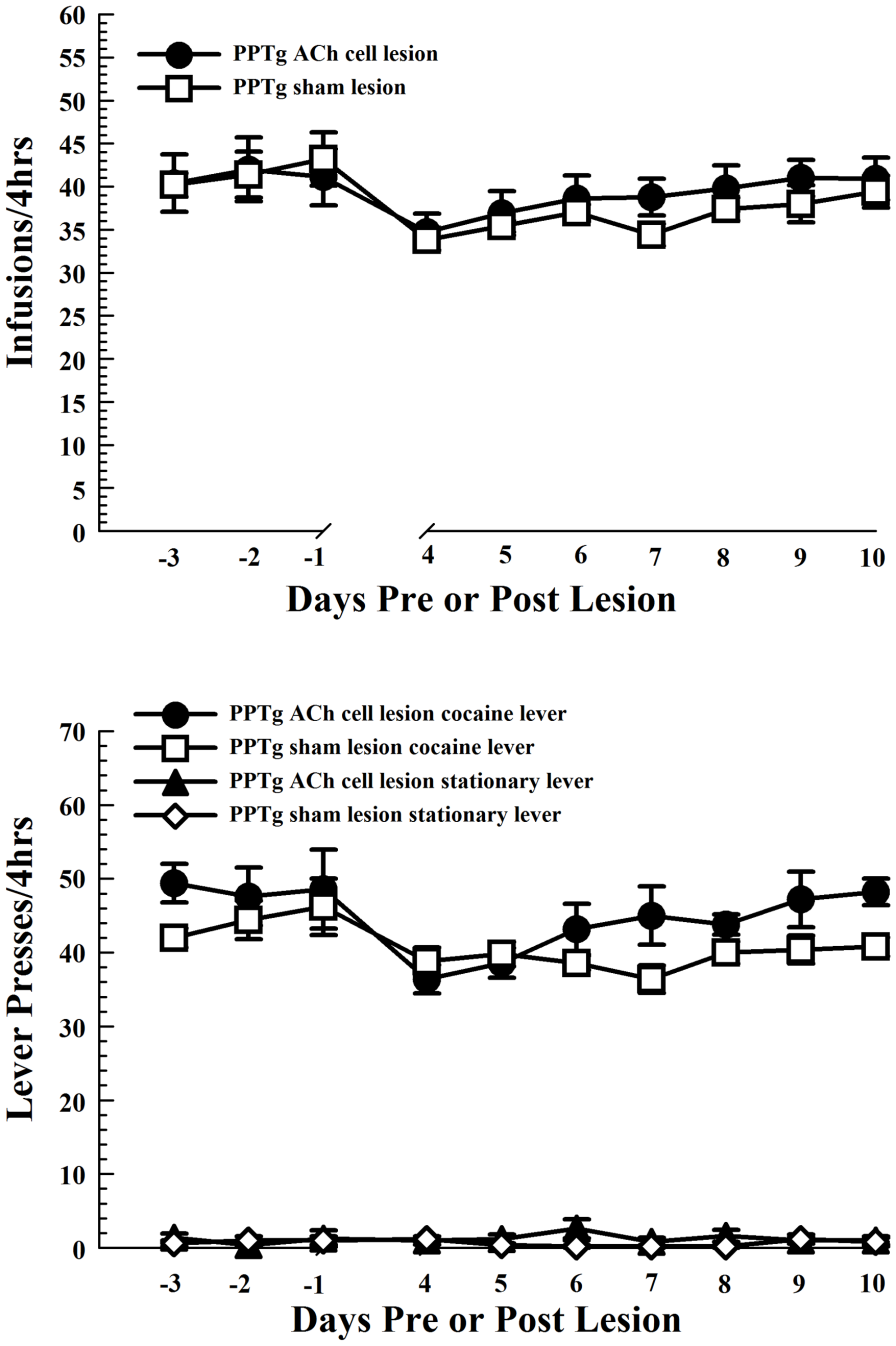
Intravenous self-administration of cocaine is not altered by bilateral PPTg cholinergic cell loss. Bilateral PPTg cholinergic cell lesions (solid circles; n = 5) did not significantly affect cocaine self-administration relative to sham lesions (open squares; n = 5), either in terms of the number of cocaine infusions in daily sessions (top) or the number of active lever presses to attain those infusions (bottom panel). All rats showed a decrease in cocaine intake on the first post-lesion testing day that returned to pre-lesion baseline levels by the third post-lesion testing day. The ability to distinguish between the retractable and stationary levers was not compromised by PPTg cholinergic cell loss. Error bars represent ± SEM.

PPTg cholinergic cell lesions failed to alter the number of either lever presses or earned cocaine infusions per session (Figure 1, main effect of LESION [F_1,8_ = 0.893, NS]). Cholinergic cell-lesioned and sham-lesioned rats showed minor but comparable decreases in cocaine intake on post-lesion test days 4 and 5 relative to the last three pre-lesion training days that recovered by post-lesion test day 6 (main effect of TIME [F_2.17, 17.34_ = 6.24, *P*<0.01], and pairwise comparisons with Tukey's HSD tests: test days 4 and 5 vs. pre-lesion training day [*P*′s<.05]). Each group responded more on the retractable than on the stationary lever throughout the testing period (main effect of LEVER [F_1,7_ = 1015.68, *P*<.0001], LEVER × LESION interaction [F_1,7_ = 1.82, NS], LEVER × TIME interaction [F_2.03,14.22_ = 1.08, NS]).

**Figure 2:**
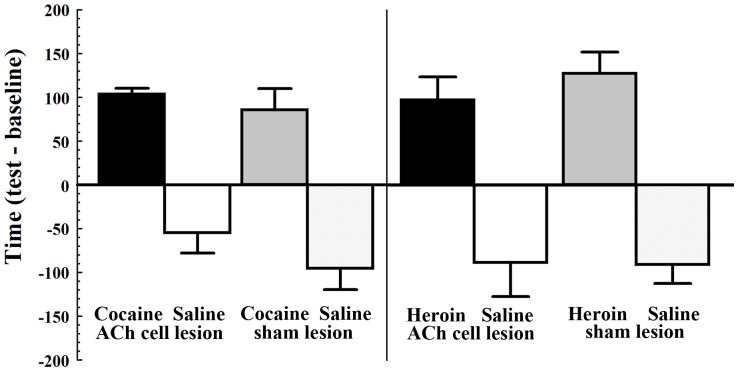
Cocaine or heroin conditioned place preferences are not altered by bilateral pedunculopontine tegmental nucleus (PPTg) cholinergic cell loss. For cocaine conditioned place preference (20 mg/kg; i.p.; left) PPTg cholinergic cell-lesioned rats (black bar; n = 4) and sham-lesioned rats (gray bar; n = 4) each showed a significant increase in the amount of time spent in the cocaine-paired chamber as a result of place conditioning. For heroin conditioned place preference (0.5 mg/kg; i.p.; right) PPTg cholinergic cell-lesioned rats (black bar; n = 6) and sham-lesioned (gray bar; n = 5) each showed a significant increase in the amount of time spent in the heroin-paired chamber as a result of place conditioning. Error bars represent ± SEM.

Development of cocaine-conditioned place preferences was also normal in PPTg cholinergic cell-lesioned rats (Figure 2, left). While cocaine established significant conditioned place preferences (F_1,6_ = 45.32, *P*<.001), they did not differ between lesion conditions.

**Figure 3:**
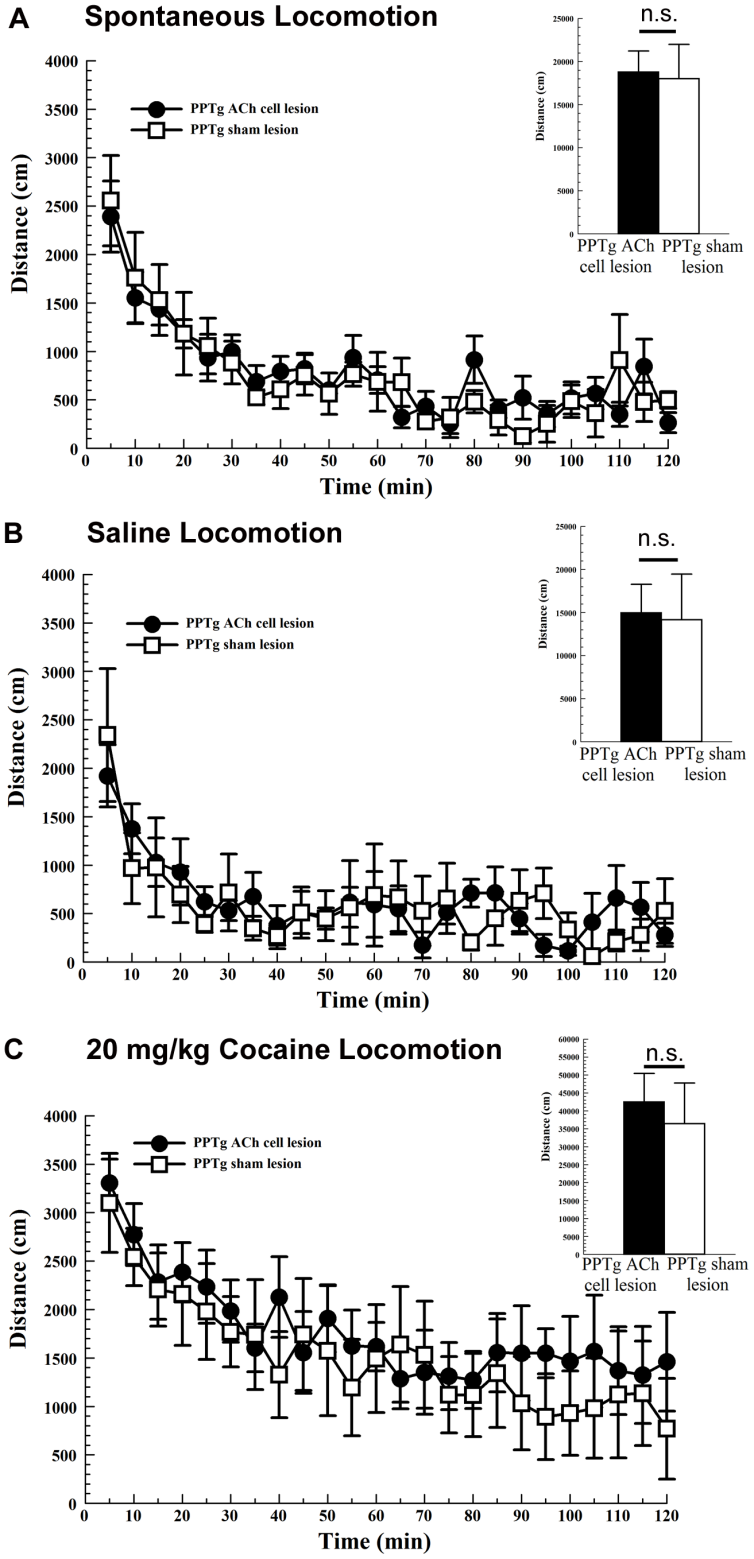
Spontaneous, saline-induced, and cocaine-induced locomotion are not altered by bilateral pedunculopontine tegmental nucleus (PPTg) cholinergic cell loss. Bilateral PPTg cholinergic cell lesions (solid circles; n = 4) did not significantly alter spontaneous locomotion (A), saline-induced locomotion (B), or cocaine-induced locomotion (C) relative to sham lesions (open squares, n = 4). Insets in A–C show total locomotion across the two-hour testing period in each of three conditions (n.s.  =  non significant). Note that locomotion was tested in the same lesioned and control rats used for the cocaine place preference (Fig. 2). Error bars represent ± SEM.

While the levels of spontaneous, saline-induced and cocaine-induced locomotion differed significantly (Figure 3, main effect of TREATMENT [F_1.06,6.36_ = 14.71, *P*<.01]) there were no significant differences between PPTg cholinergic cell-lesioned rats and sham-lesioned rats (main effect of LESION [F_1,6_ = 0.12, NS]).

### PPTg cholinergic cell loss failed to alter heroin intravenous self-administration or the establishment of heroin conditioned place preference

**Figure 4:**
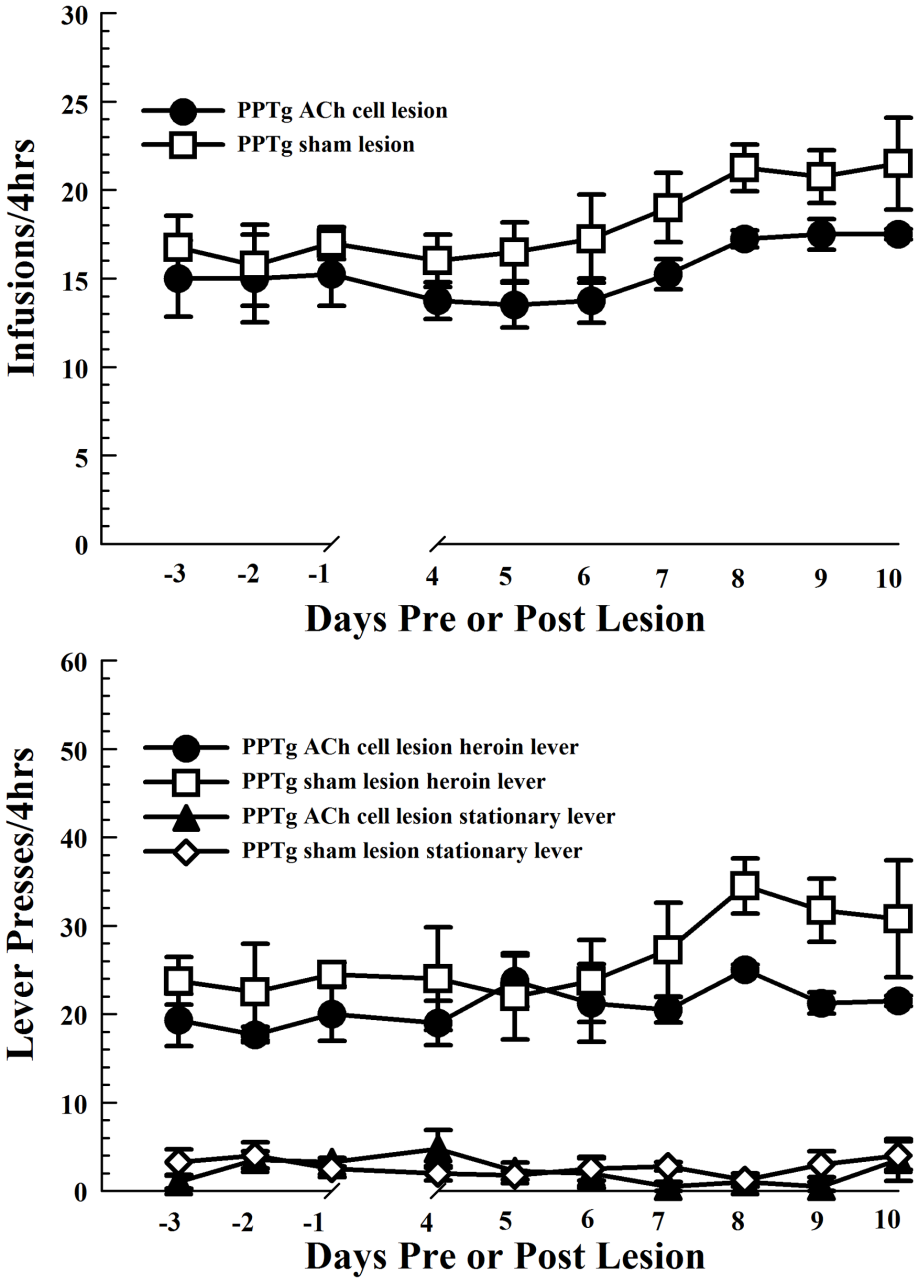
Intravenous self-administration of heroin is not altered by bilateral pedunculopontine tegmental nucleus (PPTg) cholinergic cell loss. Bilateral PPTg cholinergic cell lesions (solid circles; n = 4) did not significantly affect heroin self-administration relative to sham lesions (open squares; n = 4), either in terms of the number of heroin infusions in daily sessions (top) or the number of active lever presses to attain those infusions (bottom panel). All rats showed a slight increase in heroin intake across the post-lesion testing period. The ability to distinguish between the retractable heroin and stationary levers was not compromised by PPTg cholinergic cell loss. Error bars represent ± SEM.

PPTg cholinergic cell lesions also did not alter the number of either lever presses or earned heroin infusions per session (Figure 4, main effect of LESION [F_1,6_ = 3.29, NS]). The number of heroin infusions taken generally increased across post-lesion test days (main effect of TIME [F_9, 54_ = 3.95, *P*<0.001]; the number of infusions on days 8–10 was significantly greater than on days 1–6 in each group (pairwise comparisons with Fisher's LSD tests: days 8–10 vs. days 1–6 [*P*′s<.05]). Each group responded more on the retractable than on the stationary lever throughout the testing period (main effect of LEVER [F_1,6_ = 90.1, *P*<.0001] LEVER X LESION [F_1,6_ = 1.6, NS], LEVER X LESION X TIME interaction [F_3,18.2_ = 0.65, NS]).

Heroin-conditioned place preferences were also normal in PPTg cholinergic cell-lesioned rats (Figure 2, right). While heroin established significant conditioned place preferences (F_1,9_ = 39.5, *P*<.001), they did not differ significantly between lesion conditions.

### Histology

#### PPTg cholinergic cell loss

PPTg lesions resulted in extensive cholinergic cell loss; the ChAT-positive cell count in the lesioned rats was 2.03%±2.02% of the count in the sham-lesioned rats.

**Figure 5:**
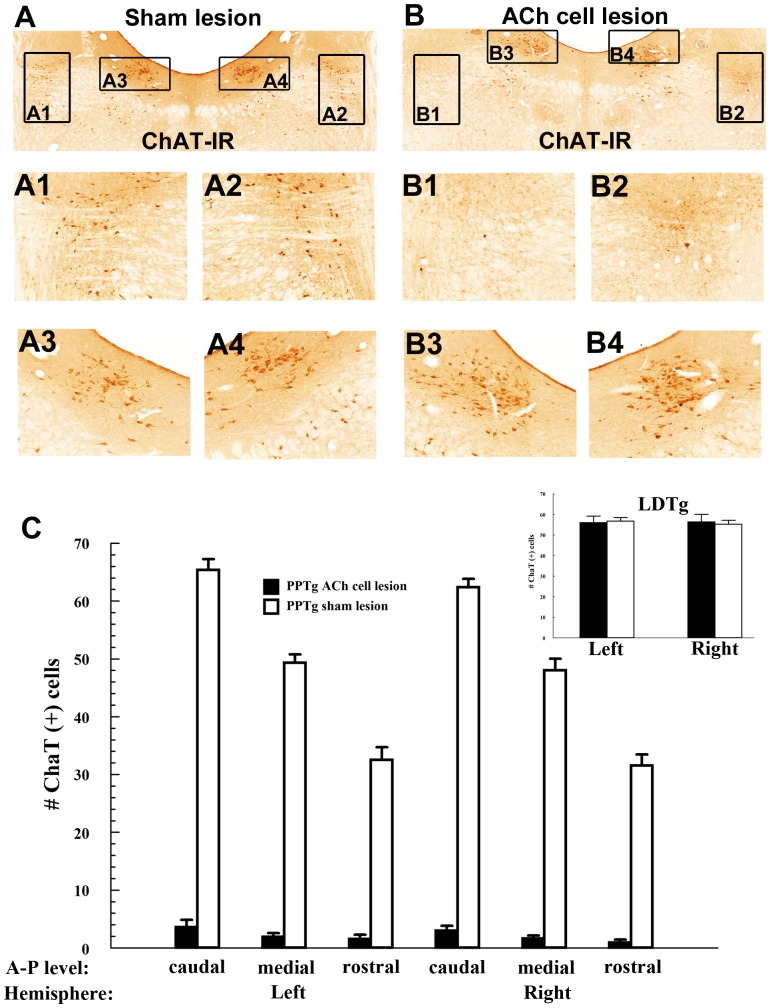
Lesions induced uniform cholinergic cell loss throughout the rostro-caudal extent of the pedunculopontine tegmental nucleus (PPTg). **Panels A–B**: Low magnification (4X) coronal PPTg sections (A-P -8.3) under bright field microscopy showing ChAT immunoreactive neurons (ChAT-IR; brown cells) following bilateral PPTg infusion of Dtx:UII (B) or vehicle (0.01 M PBS; A) from rats used in behavioral experiments. For better visualization of ChAT immunoreactivity, delimited PPTg areas in (A) and (B) are shown at higher magnification (20X) in A1 and A2 and B1 and B2, respectively. Similarly, delimited LDTg areas in (A) and (B) are shown in A3 and A4 and B3 and B4, respectively. **Panel C:** Numbers of ChAT-IR cells counted in each of two hemispheres at each of three rostro-caudal PPTg levels (A/P -8.3 caudal, A/P −7.7 medial and A/P −6.8 rostral) following cholinergic cell lesions (white bars; n = 14) or sham lesions (black bars; n = 13). Cholinergic cell loss was confined to the PPTg, as the number of ChAT immunoreactive neurons cells in the LDTg was not significantly changed (panel C, inset). Error bars represent ± SEM.

To quantify the rostro-caudal extent of PPTg cholinergic cell loss following toxin administration cholinergic cells were counted on coronal sections obtained from the rats used in heroin self-administration, heroin conditioned place preference, and cocaine conditioned place preference and locomotion experiments. In sham-lesioned, but not in cholinergic cell-lesioned rats, the number of ChAT-positive cells progressively increased from rostral to caudal levels in the PPTg (main effect of ROSTRO-CAUDAL LEVEL [F_2,102_ = 98.85, *P*<.00001], main effect of LESION [F_1,102_ = 2234.12, *P*<.00001], LESION × ROSTRO-CAUDAL LEVEL interaction [F_2,102_ = 81.50, *P*<.00001]. In lesioned rats the numbers of ChAT-positive cells was reduced at each of the three rostral to caudal levels analyzed (Figure 5; caudal PPTg: 63.88±1.19 ChAT-positive cells in sham-lesioned rats and 3.28±0.74 ChAT-positive cells in cholinergic cell-lesioned rats; medial PPTg: 48.65±1.22 ChAT-positive cells in sham-lesioned rats and 1.78±0.40 in cholinergic cell-lesioned rats; rostral PPTg: 32.04±1.43 ChAT-positive cells in sham-lesioned rats and 1.23±0.45 ChAT-positive cells in cholinergic cell-lesioned rats). The numbers of ChAT-positive cells in the adjacent LDTg of either hemisphere were not affected in PPTg cholinergic cell-lesioned rats (Figure 5C, inset).

#### Time course of cholinergic cell loss following PPTg Dtx:UII infusion

**Figure 6:**
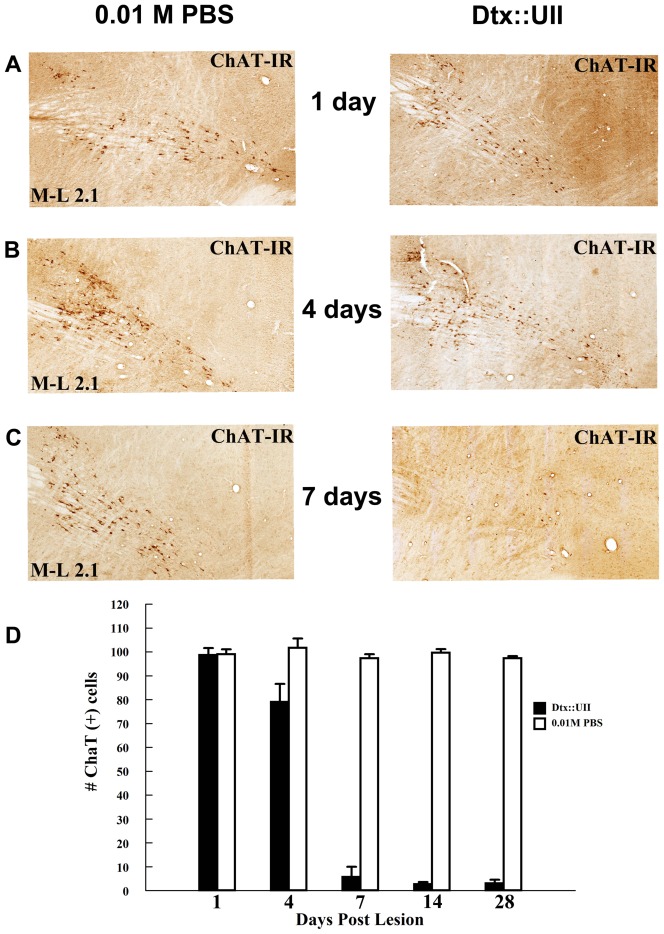
Time course of Dtx:UII-induced pedunculopontine tegmental nucleus cholinergic cell loss. **Panels A-C**: Representative sagittal PPTg sections under bright field microscopy showing choline acetyltransferase (ChAT) immunoreactivity (ChAT-IR; brown cells), 1 (A), 4 (B), and 7 (C) days following unilateral PPTg infusion of Dtx:UII (right panels) and vehicle (0.01 M PBS) infusion into the contralateral PPTg (left panels). Dtx:UII infusion induced a slight loss of PPTg ChAT-IR cells by 4 days post-infusion that became extensive by 7 days post-infusion. **Panel D:** Numbers of ChAT-IR positive cells counted at each of five time points (1, 4, 7, 14, and 28 days, n = 3 per time point) following unilateral PPTg infusion of Dtx:UII (black bars) and vehicle infusion into the contralateral PPTg (white bars) hemispheres. Dtx:UII infusion significantly reduced the number of ChAT-IR cells by 4 days post-infusion. Error bars represent ± SEM.

The numbers of ChAT-positive cells decreased with time in the lesion but not the sham lesion condition (LESION × TIME interaction [F_4,10_ = 124.31, *P*<.0001]). Most of the observed PPTg cholinergic cell loss occurred between 4 and 7 days post-infusion (Figure 6A). There was no significant cholinergic cell loss in the first 24 hours but significant cholinergic cell loss by 4 (*P*<0.001), 7, 14 and 28 (*P*′*s*<.000001) days after the infusion (Figure 6B).

#### Specificity of cholinergic cell loss following PPTg Dtx:UII infusion

**Figure 7:**
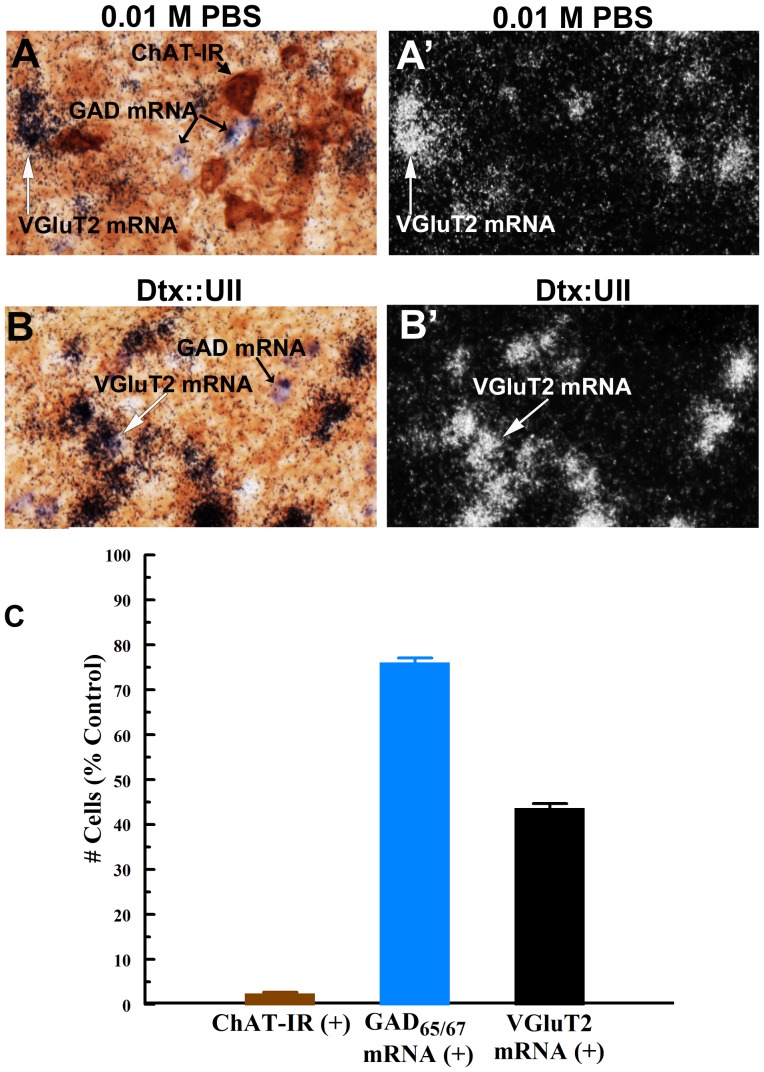
Dtx::UII infusion induced loss of PPTg glutamatergic and GABAergic cells. **Panels A and B:** Representative sagittal PPTg sections showing ChAT immunoreactive neurons (ChAT-IR; brown cells) and GAD_65/67_ mRNA (purple cells) under bright field microscopy (left) and VGluT2 mRNA as white aggregates under epiluminescence microscopy (right) 14 days following unilateral PPTg infusion of vehicle (0.01 M PBS; A and A′) and Dtx:UII (B and B′) into the contralateral PPTg. **Panel C:** Numbers of cholinergic (ChAT), glutamatergic (VGluT2), and GABAergic (GAD_65/67_) cells following unilateral PPTg infusion of Dtx:UII (left panels), expressed as a percentage of the number of each cell type counted in the contralateral hemisphere following vehicle infusion (n = 3). Unilateral PPTg Dtx::UII infusion induced significant loss of cholinergic, glutamatergic, and GABAergic cells. Error bars represent ± SEM.

Fourteen days after unilateral PPTg Dtx::UII infusion there was significant glutamatergic and GABAergic cell loss in addition to significant cholinergic cell loss (56.7±1.35% glutamatergic cell loss [t_2_ = −41.85, p<.001], 24.29±1.33% GABAergic cell loss [t_2_ = 57.06, p<.001], and 97.93±0.61% cholinergic cell loss [t_2_ = −162.42, p<.0001]). The relative loss of cholinergic cells was significantly greater than the loss of either glutamatergic or GABAergic cells (p′s<.0001; Figure 7).

## Discussion

Despite previous evidence implicating cholinergic input to the VTA in cocaine reward and in brain stimulation reward, near-total loss of PPTg cholinergic neurons failed to significantly alter self-administration of cocaine or heroin or to alter the place preferences established with these drugs; the present data suggest that the rewarding effects of neither cocaine nor heroin depends critically on the cholinergic contribution of the PPTg. Given the lack of lesion effects on heroin conditioned place preference or self-administration, it would appear that it is damage to non-cholinergic PPTg neurons that is responsible for the effects of nonspecific PPTg lesions on the acquisition of these behaviors [21], [22].

The evidence implicating acetylcholine in the rewarding effects of cocaine is, first, that self-administered cocaine elevates extracellular VTA acetylcholine levels and, second, that blocking VTA cholinergic receptors causes compensatory increases in cocaine-taking [8]. The evidence implicating acetylcholine in opiate reward is, first, that blocking VTA cholinergic receptors attenuates the acquisition of morphine conditioned place preference [23] and, second, that the acquisition of morphine conditioned place preference is reduced in M_5_ muscarinic acetylcholine receptor knockout mice [24].

Extracellular levels VTA acetylcholine levels are elevated during extinction responding for cocaine in rats and inhibition of acetylcholine degradation in the VTA reinstated extinguished cocaine seeking in rats [8]. Cholinergic receptor blockade in the VTA decreases cocaine priming-induced reinstatement [25]. Acquisition of a cocaine conditioned place preference is assessed by the extent to which a rat approaches a set of environmental cues that were previously paired with cocaine. We expected that the acquisition of cocaine conditioned place preference would be compromised following PPTg cholinergic cell loss, but this prediction was clearly not supported by the data.

The lack of effect of total loss of PPTg cholinergic neurons raises two possibilities for future studies. First, it may be that the LDTg is the important source of VTA acetylcholine levels. PPTg and LDTg each contain independent populations of cholinergic, glutamatergic, and GABAergic neurons that project to the ventral midbrain, and while the projections of cholinergic neurons have not been selectively quantified the general rule is that the more medial LDTg projects more strongly to the more medial VTA and the more lateral PPTg projects more strongly to the more lateral substantia nigra [9]. It is possible that the LDTg is more important than PPTg in reward function or it may be that the LDTg can compensate in some way for loss of PPTg input to the reward system.

The lack of effects was not due to ineffective lesions. The injections caused nearly complete PPTg cholinergic cell loss and avoided loss of closely adjacent LDTg cholinergic cells. The observed glutamatergic and GABAergic cell loss may be secondary to the primary loss of acetylcholine neurons, but it may be that the Dtx:UII conjugate has some degree of toxic action even when it does not bind to the urotensin-II receptor.

In summary, our data show that PPTg cholinergic cells are not critical for the rewarding effects of either cocaine or heroin.

## Materials and Methods

### Animals and Surgery

Forty-two male Long-Evans rats (Charles River, Raleigh, NC) weighing 275–325 grams at the time of surgery were used for behavioral experiments. An additional 15 male Long-Evans rats were used to assess the time course and specificity of cholinergic cell loss following toxin administration. All rats were individually housed under a reverse light-dark cycle (12/12, lights off at 8 am) with free access to food and water throughout all experiments. All experiments were performed in accordance with the guidelines outlined in the National Institutes of Health Guide for the Care and Use of Laboratory Animals and were approved by the Animal Care and Use Committee of the NIDA Intramural Research Program.

### Drugs and neurotoxin

Ketamine, (-)-cocaine hydrochloride, and 3,6-diacetylmorphine hydrochloride (heroin) were obtained from the NIH. Cocaine and heroin were each dissolved in 0.9% sterile saline. Diphtheria toxin conjugated to urotensin-II (Dtx::UII) [20], [26] was obtained from One World Biotech (Warminister, PA).

### Surgery

For all surgical procedures, rats were anesthetized first with a combination of ketamine and xylazine (57 mg/kg and 9 mg/kg i.p., respectively). Anesthesia was then maintained during the surgery with isoflurane (2–3% in 1 L/min Oxygen). For studies involving time course analysis and specificity of cholinergic cell loss or conditioned place preference, rats underwent a single surgery during which Dtx::UII or vehicle was injected into the PPTg. For studies involving intravenous drug self-administration, rats were first implanted with a jugular vein catheter and received PPTg Dtx::UII or vehicle injections after completion of self-administration training.

#### PPTg Dtx::UII infusion

Each rat was anesthetized and secured in a stereotaxic apparatus (David Kopf; Tujunga, CA) and three injections of 200 nl each of either Dtx::UII (42 nanograms protein per injection; 126 nanograms protein total per hemisphere) or 0.01 M phosphate-buffered saline (vehicle) were made into each PPTg using a syringe controlled by an automated infusion pump (UMP 3; World Precision Instruments; Sarasota, FL) attached to one of the stereotaxic manipulator arms. The injector needle was lowered into each PPTg from the rear along an angled trajectory of 35° from vertical. The three injections per hemisphere were aimed at the following coordinates according to the rat brain atlas of Paxinos & Watson [27] relative to lambda: 1) A-P +0.6, M-L ±2.1, D-V −6.9, 2) A-P +0.3, M-L ±2.1, D-V −6.5, 3) A-P +0.0, M-L ±2.1, D-V −6.2. The needle tip was lowered along the angled trajectory to the most rostral coordinate and the first injection was made. The needle was then retracted to the second, more caudal coordinate and the second injection was made. The needle was then retracted to the third, most caudal coordinate and a third injection was made. Following each infusion, the injector was left in place for five minutes to allow for diffusion of the injected solution form the needle tip.

#### IV catheter implantation

Each of the 23 rats used for drug self-administration was implanted with an intravenous microranathane catheter (Braintree Scientific; Braintree, MA) inserted into the right external jugular vein. Catheter tubing was attached to a cannula adaptor backmount assembly at the other end. The backmount assembly exited the animal's back just caudal to the scapulae. Catheters were flushed daily with heparin (10 USP/ml in sterile saline), containing gentamicin (0.08 mg/ml).

#### Time course analysis and specificity of PPTg cholinergic cell loss

Fifteen rats were used to assess the time course and specificity of PPTg cholinergic cell loss following toxin infusion. These rats each received a unilateral PPTg Dtx::UII infusion, and a vehicle infusion into the PPTg of the opposite hemisphere. Following the infusions, the rats were returned to their home cages and three rats were then euthanized at each of five time points (24 hrs, 4 days, 7 days, 14 days, or 28 days). Their brains were examined for the extent and specificity of cholinergic cell loss (see below).

### Drug self-administration

#### Drug self-administration apparatus

Operant conditioning chambers (Med Associates; Georgia, VT), measuring 25×27×30 cm, were used for cocaine or heroin self-administration studies. Each chamber was housed within a sound-attenuating enclosure equipped with a fan, which provided both ventilation and a constant source of noise during behavioral testing. Each chamber was equipped with a retractable lever and a stationary lever, a red house light, illuminated throughout the session, and a white cue light located above the retractable lever, illuminated whenever an infusion was earned.

#### Drug self-administration procedure

Daily 4-hour intravenous drug self-administration training sessions began 7–10 days after i.v. catheter implantation and lasted for 13–15 days. The rats were transported daily to their individual operant chambers and their backmount catheters were connected to an infusion line which was attached to a liquid swivel that allowed for free movement within the operant chamber. The beginning of each training session was marked by illumination of the red house light and insertion of the retractable lever into the operant chamber. No priming infusions were given. Depending on the experiment, separate groups of rats each were allowed to self-administer cocaine (1.0 mg/kg/infusion, i.v.; n = 15) or heroin (0.1 mg/kg/infusion, i.v.; n = 8) under a fixed ratio 1 schedule of reinforcement with a 20-s time-out period accompanied by illumination of a white cue light located above the retractable lever. Presses on the retractable lever during the time-out period were recorded but were not reinforced. Presses on the stationary lever had no programmed consequence. During the training phase rats were required to reach a criterion of three consecutive days of stable responding defined as less than 10% variability across days and a minimum of 30 cocaine or 10 heroin infusions per 4-hour session. The rats were then randomly assigned to either PPTg Dtx::UII (ACh cell lesion) or vehicle (sham lesion) treatment groups and underwent the intracranial toxin injection procedure. Self-administration testing was resumed four days after PPTg Dtx::UII or vehicle injections and continued for an additional 7 days of training.

### Conditioned place preference

#### Conditioned place preference apparatus

Three-chamber conditioned place preference boxes (Med Associates; Georgia, VT) were used for experiments involving either heroin or cocaine conditioned place preference. Each conditioned place preference box consisted of two conditioning chambers (21×21×28 cm) connected by a neutral start chamber (21×21×12.5 cm). One of the conditioning chambers was black, had a rod floor, and was more strongly lighted, while the other was white, had a mesh floor, and was more dimly lighted so as to balance chamber preference. The neutral chamber was gray and had a smooth Plexiglas floor.

#### Conditioned place preference training

Place preference conditioning was initiated 10 days after PPTg cholinergic cell or sham lesions. Nineteen rats were each given an initial 30-min habituation session, during which they were free to explore the entire place preference apparatus. During a 15-min baseline test given one week later the rats were free to explore the entire apparatus for a period of 15 minutes. The times spent in each of the three chambers were recorded. Conditioning began one day after the baseline test. For cocaine place preference conditioning, 8 rats received injections of cocaine (20 mg/kg, i.p.) or saline (1 ml/kg, i.p.) and were confined to the assigned conditioning chambers for a period of 20 minutes. For heroin conditioned place preference, 11 rats received injections of heroin (0.5 mg/kg, i.p.) or saline (1 ml/kg, i.p.) and were confined to the assigned conditioning chambers for a period of 30 minutes. In either case, each rat received four drug and four saline injections, on alternate days, for a total of eight conditioning days. In each of the cocaine and heroin groups, the assignment of drug and saline to the two conditioning chambers was counterbalanced, with half of the rats receiving drug injection prior to placement in the dimly lighted white chamber and the other half prior to placement in the more brightly lighted black chamber. One day after the final conditioning session, each rat was again given a 15-min preference test during which it was free to explore the entire apparatus. The times spent in each of the three chambers were recorded.

### Cocaine-induced Locomotion

One week after the completion of cocaine conditioned place preference the extent of spontaneous and cocaine-induced locomotion was measured in the same 4 PPTg-lesioned and 4 control rats used for the cocaine conditioned place preference experiment described above. Locomotor activity was measured using open field enclosures (Accusan, Columbus, OH) equipped with photobeams and housed within sound-attenuating cubicles. On the first day of testing each rat was placed in an open-field enclosure for 2 hrs. On the second and third days of testing each rat received either cocaine (20 mg/kg; i.p.) or saline (1 ml/kg; i.p.) and was placed in an open-field enclosure for 2 hrs. The order of cocaine and saline injections across testing days 2 and 3 was counter-balanced across rats.

### Histology

#### Tissue Preparation

Following completion of behavioral testing or at the appropriate time point following unilateral toxin infusion, each rat was deeply anesthetized with a mixture of pentobarbital (30 mg/kg, i.p.) and chloral hydrate (140 mg/kg, i.p.). It was then transcardially perfused with 0.1 M phosphate buffer (PB) followed by 4% (W/V) paraformaldehyde in 0.1 M PB, pH 7.3, and its brain was removed and left in 4% paraformaldehyde for 2 hours at 4°C and then transferred to a 18% sucrose solution in PB. Sagittal or coronal sections (20 µm in thickness) were prepared for each rat, depending on the experiment. Sagittal sections were prepared for experiments involving time course analysis of cholinergic cell loss, and cocaine intravenous self-administration, while coronal sections were prepared for experiments involving heroin intravenous self-administration, cocaine conditioned place preference, and heroin conditioned place preference.

#### Immunocytochemistry

Twenty-seven brains were cut in the coronal and 25 brains in the sagittal plane. The sections were rinsed with 3×10 min PB, followed by 15 minutes incubation in 0.3% H_2_O_2_, followed by 1 hour incubation in PB supplemented with 4% bovine serum albumin (BSA) with 0.3% Triton X-100. The sections were then incubated with anti-ChAT goat polyclonal antibody (Chemicon; Temecula, CA, AB144P; dilution 1∶100) overnight at 4°C. After rinsing with 3×10 min PB, the sections were incubated for 1 hour at room temperature in a 1∶200 dilution of biotinylated secondary antibody (Vector Laboratories; Burlingame, CA), rinsed with PB, and incubated with avidin-biotinylated horseradish peroxidase for 1 hour. The sections were then rinsed and the peroxidase reaction was developed with 0.05% 3, 3-diaminobenzidine-4 HCl (DAB) and 0.03% hydrogen peroxide (H_2_O_2_). Finally, the sections were mounted on gelatin-coated slides and air-dried for inspection.

#### Double *in situ* hybridization for the vesicular glutamate transporter 2 (VGluT2) and glutamic acid decarboxylase (GAD_65/67_) in combination with ChAT immunocytochemistry

To assess the specificity of cholinergic cell loss following unilateral PPTg toxin infusion sections were processed for expression of vesicular glutamate transporter 2 (VGluT2) messenger RNA, glutamic acid decarboxylase (GAD_65/67_) mRNA and ChAT protein as described by Wang and Morales [28].

#### Cell counting

Processed sections were viewed, analyzed, and photographed under bright field or epiluminescence microscopy using Surveyor with Turboscan software (Objective Imaging, Cambridge, UK) at 20X. For each of the 15 rats used to determine the time course of PPTg cholinergic cell loss and for each of the rats used in the cocaine self-administration experiment one representative sagittal section showing the PPTg was chosen from the hemisphere previously injected with vehicle. For each of these sections the boundaries of the PPTg were determined based on the extent of ChAT staining and outlines were traced accordingly. The number of ChAT-positive cells was counted within these boundaries. The tissue collected 14 days after unilateral Dtx::UII infusion was also processed for simultaneous detection of VGluT2 and GAD_65/67_ mRNA and ChAT protein to assess the specificity of cholinergic cell loss. In these cases, the number of PPTg VGluT2-positive and GAD_65/67_-positive nuclei were also counted. In either case, the outlines of several adjacent brain nuclei were traced in addition to the outlines of the PPTg. Matched sections were then chosen from the contralateral hemisphere previously injected with Dtx::UII based on these structures and the overall shape of the sagittal section. The outlines created from the sham-lesioned hemisphere were superimposed, and the number of ChAT-positive cells were counted, and, in cases involving triple labeling of tissue, the number of VGluT2-positive and GAD_65/67_-positive nuclei were also counted within the previously determined PPTg boundaries. The percentage of cell loss for each cell population in the lesioned hemisphere, relative to the sham-lesioned hemisphere, was analyzed.

For those rats used in cocaine CPP, cocaine locomotion, heroin CPP, and heroin self-administration experiments lesions were evaluated using coronal sections as this allowed for better quantification of the rostro-caudal extent of cholinergic cell loss. In these cases one representative coronal section was chosen from each sham-lesioned control rat showing each of the rostral (A-P −6.8), medial (A-P −7.7) and caudal (A-P −8.3) portions of the PPTg. The boundaries of the PPTg were determined in sham-lesioned control rats based on the extent of ChAT staining and the outlines were traced accordingly. The number of ChAT-positive cells was counted within these boundaries. In addition to the outlines of the PPTg, the outlines of several adjacent brain nuclei were traced. Based on these structures, matched sections were then chosen from Dtx:UII cholinergic cell-lesioned rats. The outlines created from the sham-lesioned control rats were superimposed onto these matched sections and the numbers of ChAT-positive cells were counted within the previously determined PPTg boundaries. In sections showing the most caudal portions of the PPTg, the numbers of LDTg ChAT-positive cells in each hemisphere were also counted for each rat.

### Data Analysis

Data were analyzed using between-within subjects Analysis of variance (ANOVA). Where applicable, significant main effects were followed with Fisher's LSD post-hoc tests. The degrees of freedom for tests involving within-subjects factors and interactions between within and between-subjects factors were adjusted using Greenhouse-Geisser corrections when necessary. For the cocaine self-administration experiment three rats developed catheter problems during the pre-lesion training period. In addition, one cholinergic cell-lesioned rat died during the post-lesion recovery period and one sham-lesioned rat developed a catheter problem during the post-lesion testing period. Statistical analysis of cocaine self-administration data was thus performed on the remaining 5 cholinergic cell-lesioned and 5 sham-lesioned rats.
